# Organoid-based Models to Study the Role of Host-microbiota Interactions in IBD

**DOI:** 10.1093/ecco-jcc/jjaa257

**Published:** 2020-12-20

**Authors:** Martina Poletti, Kaline Arnauts, Marc Ferrante, Tamas Korcsmaros

**Affiliations:** 1 Earlham Institute, Norwich Research Park, Norwich, UK; 2 Quadram Institute, Norwich Research Park, Norwich, UK; 3 Department of Chronic Diseases, Metabolism and Ageing [CHROMETA], Translational Research Center for Gastrointestinal Disorders [TARGID], KU Leuven, Leuven, Belgium; 4 Department of Development and Regeneration, Stem Cell Institute Leuven [SCIL], KU Leuven, Leuven, Belgium; 5 Department of Gastroenterology and Hepatology, University Hospitals Leuven, KU Leuven, Leuven, Belgium

**Keywords:** Inflammatory bowel disease, microbiota, organoids, *in vitro* models

## Abstract

The gut microbiota appears to play a central role in health, and alterations in the gut microbiota are observed in both forms of inflammatory bowel disease [IBD], namely Crohn’s disease and ulcerative colitis. Yet, the mechanisms behind host-microbiota interactions in IBD, especially at the intestinal epithelial cell level, are not yet fully understood. Dissecting the role of host-microbiota interactions in disease onset and progression is pivotal, and requires representative models mimicking the gastrointestinal ecosystem, including the intestinal epithelium, the gut microbiota, and immune cells. New advancements in organoid microfluidics technology are facilitating the study of IBD-related microbial-epithelial cross-talk, and the discovery of novel microbial therapies. Here, we review different organoid-based *ex vivo* models that are currently available, and benchmark their suitability and limitations for specific research questions. Organoid applications, such as patient-derived organoid biobanks for microbial screening and ’omics technologies, are discussed, highlighting their potential to gain better mechanistic insights into disease mechanisms and eventually allow personalised medicine.

## Introduction

Both forms of inflammatory bowel disease [IBD], Crohn’s disease [CD] and ulcerative colitis [UC], are thought to be driven by environmental factors in genetically susceptible individuals, resulting in an exacerbated immune response towards components of the gut microbiota.^1^ However, the underlying mechanisms are not yet completely understood. In particular, whether the loss of tolerance towards the microbiota is a cause or consequence of the disease, and what the exact effect is of the interactions between intestinal epithelial cells and the dysbiotic microbiota, remain unclear.

Growing evidence suggests that the cross-talk between the luminal microbiota and the intestinal epithelium plays a key role in the onset of IBD.^2^ The intestinal microbiota interacts with the epithelium of the gut through metabolites or other released factors,^3–5^ and contributes to the intestinal barrier functions and integrity,^3,4^ modulates the host immune system,^6^ and prevents colonisation of pathobionts.^6^ Microbial metabolites such as short chain fatty acids [SCFAs], produced by microbial fermentation of dietary fibre in the gut, also serve as an energy source^7^ and as immunomodulators.^8^

Alterations in microbiota composition and homeostasis [‘dysbiosis’] have been observed in both forms of IBD,^9–11^ overall with a decrease in gut microbial diversity and a shift in the balance between commensal and pathobionts.^12^ For example, a reduction in the Firmicutes phylum and an increase in Proteobacteria has repeatedly been observed.^13^ This change in microbiota is also associated with a shift in fermentations products, such as SCFAs.^14^ For instance, a decrease in butyrate-producing species has been observed in UC.^15^

In addition, the intestinal epithelium plays a key role in IBD.^16–18^ This is illustrated by the presence of several IBD-associated genetic defects associated with bacterial sensing [*NOD2*], inflammation [*IL-23R*], autophagy [*ATG16L1*], endoplasmic reticulum stress, and epithelial barrier [*HNF4α*, *CDH1*, *MEP1A*, *CARD15*, *ATG16L1*] functions in epithelial cells.^19,20^ In IBD, intestinal epithelial cell dysfunction promotes increased epithelial barrier dysfunction and permeability.^21–23^ In a healthy condition, the presence of a single mucus layer in the small intestine and bi-layered mucus structure in the colon—with an impermeable inner layer and a permeable outer layer—prevents intestinal epithelial cells from being in direct contact with commensal bacteria and potential pathogens.^24^ In IBD, changes in the integrity of the inner layer due to defects in mucus production result in intestinal barrier dysfunction.^25^ As a consequence, increased bacterial and metabolite translocation are observed,^26,27^ overall resulting in immune cell activation and inflammation.^28^ Pro-inflammatory factors released in the intestinal mucosa during active disease progressively damage the epithelial layer.^26,27^ Despite the importance of dysfunctional epithelial-microbial cross-talk in both types of IBD, the exact cause and mechanism of these interactions are yet not completely understood.

Strong evidence supports a causal role for the gut microbiota in IBD development.^29^ Using mouse colonic inflammation models, it was observed that only conventionally raised mice developed inflammation, whereas germ-free mice [with no microbiota] did not.^30^ Furthermore, transferring the gut microbiota from a colitis mouse model to a wild-type mouse resulted in the induction of inflammation.^31^ In addition, recurrence of CD in patients who had undergone an [ileocolonic] resection could be prevented in the absence of faecal stream [and thus microbiota],^32^ and triggered in the presence of intestinal fluids.^32,33^

Because of the strong relationship between dysbiosis and IBD, probiotics formulations,^34,35^ SCFAs,^8,36^ and faecal microbiota transplantation [FMT]^37,38^ have been explored for their ability to restore the microbiota composition and inflammatory status associated with IBD.^39^ However, understanding of the effectiveness and mechanisms is still lacking.^40,41^ It remains unclear which bacteria [single, or complex mixtures] are required to induce and maintain remission in IBD patients. Moreover, given the heterogeneous nature of IBD, it is likely that a microbial intervention will need to be tailored for each individual to reduce dysbiosis and promote immune tolerance and homeostasis in IBD.

**Figure 1.**
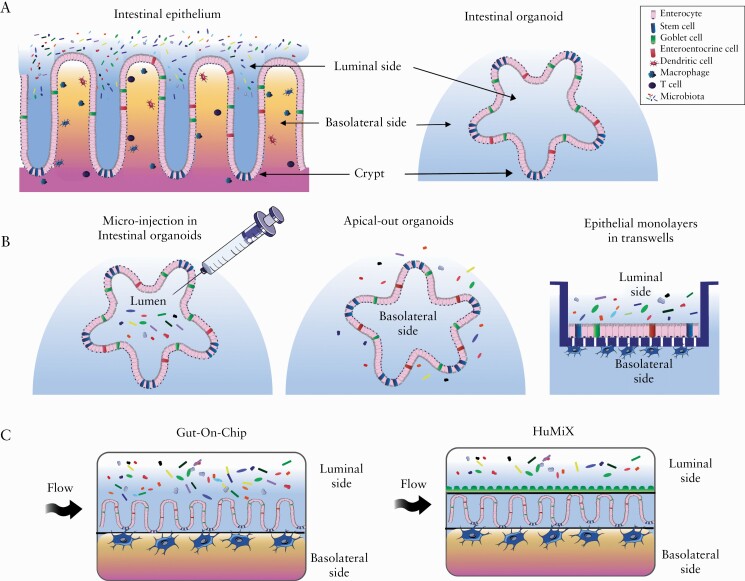
[A] The intestinal epithelium and the organoid model. [B] 3D organoids and organoid-derived models. [C] Microfluidics models.

Understanding the complex interactions between the microbiota and the intestinal epithelium, and how they impact on the individual’s disease onset and progression, is essential. To achieve this, specific *in vitro* systems mimicking the intestinal epithelium in a representative model are needed. The development of intestinal organoids—three-dimensional [3D] *in vitro* models of the human intestinal epithelium—has represented a major advancement in the field **[**Figure 1A]. The use of organoids to study host-microbe interactions has been previously reviewed.^42^ However, novel exciting organoid systems have brought many new possibilities especially relevant for IBD research.

**Figure 2.**
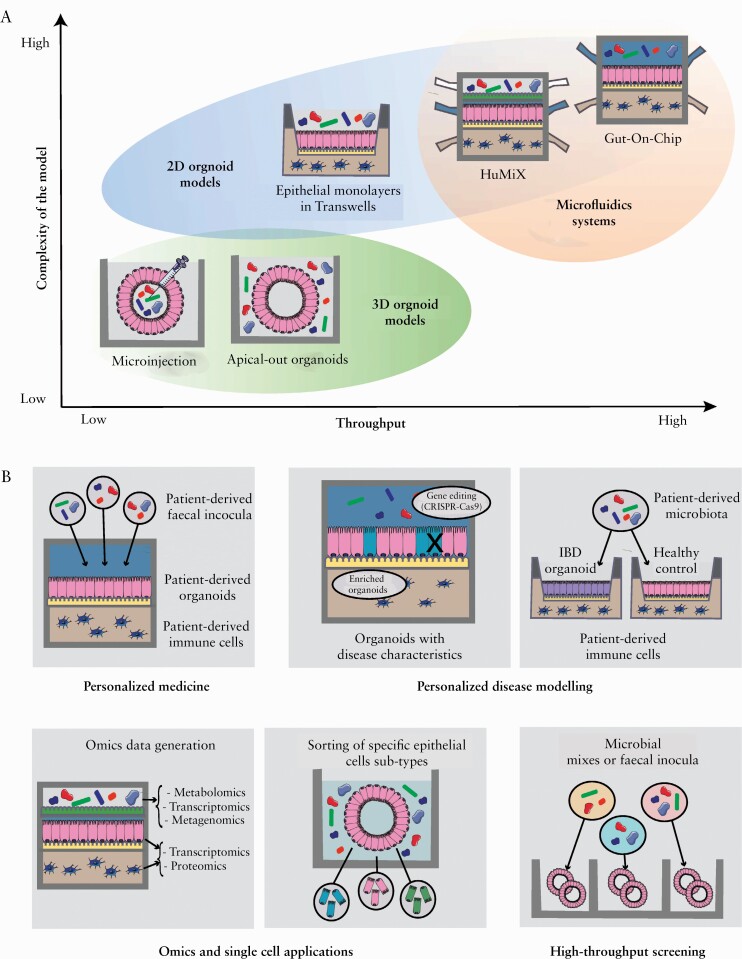
Organoid-based models, [A] evolution; [B] and applications.

In this review, we present the evolution of the organoid [derived] models available [Figure 2A], from basic 3D organoid models and their 2D counterparts, to more complex microfluidic-based systems [Figure 1B-C]. The suitability of these systems will be benchmarked for their ability to model the complex interactions happening in IBD, specifically host-microbiota interactions, for specific research questions **[**Table 1**]**. Finally, we present the main applications and future trends **[**Figure 2B].

**Table 1.** Benchmarking the different organoid models for host-microbiota studies in IBD.

Model complexity	In the Gut-Chip, peristalsis allows higher epithelial differentiation resulting in better mucus production. In HuMiX, this is achieved with an artificial mucus layer. The introduction of organoids [possible in all models], and other cell types [anaerobe Transwell, HuMiX, anaerobic Gut-Chip] can make the model more complete
Type of inoculum	The anaerobic Gut-Chip, HuMiX, and microinjection are preferred when co-culturing complex microbiota, whereas other models are more suitable for single bacteria or metabolites
Anaerobiosis	Strict anaerobes can be introduced within HuMiX, the anaerobic Gut-Chip [longer assays], or 3D organoids and the anaerobe Transwell [shorter assays]. Conversely, apical-out organoids and Transwells can only sustain the growth of facultative anaerobes for short-term assays
Nature of the interaction	Transwells and apical-out organoids provide a direct microbial-epithelial [villus] interface, which is relevant in IBD where the mucus barrier is often disrupted. Instead, because HuMIX presents a physical separation between microbes and epithelial cells, it is more suitable to study metabolites
Co-culture time	Static systems such as Transwells, microinjection, apical-out organoids, can sustain co-cultures for short times [<24 h], whereas the constant medium flow within the anaerobic Gut-Chip allows co-cultures for up to 5 days. HuMiX has been used for 24 h only, but could accommodate longer assays
Outcome measures	3D organoids and Transwells allow high-throughput experiments, and can be used to evaluate transcriptional gene expression. In contrast, microfluidics systems such as the anaerobic Gut-Chip and HuMiX allow selected conditions to be studied in depth, for long-term transcriptional and metabolic profiling
Availability/cost	Apical-out organoids, microinjection, and Transwells models allow low-cost and widely replicable assays. Conversely, the anaerobic Transwell model, HuMiX, and the anaerobic Gut-Chip are less accessible, and their associated cost is higher

IBD, inflammatory bowel disease.

## 2. Patient-derived Organoids to Investigate Host-microbiota Interactions

The study of the host-microbiota cross-talk *in vivo* is limited by the relative inaccessibility of the human digestive tract. Faecal samples can be easily obtained, but intestinal aspirates and biopsies are less accessible in adequate numbers.^43^ Manipulation and precise control over experimental variables in human studies, including host and microbial genetics, also remains a difficult task. The effects of the microbiota are location [eg, sites of inflammation] and time dependent,^11^ and *in situ* spatio-temporal measurements of microbiota and host responses at sufficient resolution are not currently possible in humans.

Microbiota studies have been generally carried out in mice,^44^ thanks to their similar gastrointestinal architecture, and because they allow an easier sample collection and better control over both diet and genetics.^45^ However, mouse models are unable to fully represent the physiological conditions of the human gut,^46^ nor can they perfectly recapitulate human disease.^47^ Alternatively, colonic cell lines such as Caco-2, T84, and HT-29 have also been employed.^48–50^ Yet, cell lines do not reflect tissue heterogeneity or location-specific characteristics [duodenum, colon, etc].^51^

Patient-derived intestinal organoids represent a unique tool to study host-microbiota cross-talk, overcoming some of the disadvantages associated with human studies, mouse models, and cell lines. Furthermore, organoids allow repeated experiments and maintain organ, disease, and patient characteristics.^52,53^

### 2.1. Patient-derived intestinal organoids

Intestinal organoids are 3D *in vitro* epithelial structures derived from primary tissue, capable of self-renewal and self-organisation, recapitulating the architecture and function of the human gastrointestinal tract.^54^ Intestinal organoids can be derived either from adult human intestinal stem cells [hASCs], hASCs containing crypts,^55^ or human induced pluripotent stem cells [iPSCs].^56^ Stem cells are embedded in a cell culture matrix, mimicking the extracellular matrix [ECM], which allows the self-assembly of a crypt-villi structure including the luminal surface of epithelium projected towards the centre of the organoid and the basolateral side in contact with the ECM and surrounding medium **[**Figure 1A**]**.

Whereas ASC-derived organoids can be differentiated into the different intestinal epithelial cell types,^55^ iPSC-derived organoids also contain the intestinal mesenchyme.^56^ Furthermore, iPSC-derived organoids have a low grade of maturation and therefore resemble more fetal tissue compared with ASC-derived organoids—so they are less favourable to model adult tissue biology. Organoids derived from human biopsies maintain the crypt-villi structure of the intestine, and retain the genetic background and transcriptional and epigenetic characteristics of the intestinal segment [duodenum, jejunum, ileum] they were derived from.^57–60^ Organoids from IBD patients maintain disease-specific characteristics,^61^ including altered gene expression profiles associated with absorptive and secretory functions.^62^ The acute transcriptional inflammatory phenotype is lost during organoid culture but can be re-induced after inflammatory stimulation,^57^ and an accumulation of somatic mutations in inflamed UC mucosa has been associated with disease duration.^63^

Contrary to intestinal cell lines, differentiated intestinal organoids contain all the epithelial cell lineages populating the intestinal crypt,^55^ including rare cells [enteroendocrine, tuft, or M cells]^64–66^ or cells that could not be previously cultured *in vitro* [eg, Paneth cells].^67^ By manipulating the culture conditions, organoids can also be maintained in a non-differentiated status, containing mainly intestinal stem cells and progenitor cells.^52^ In this way, IBD organoids represent a unique tool to investigate the cell-type specificity of host-microbial interactions in a patient-specific manner.

Below, we will introduce the different organoid models that enable us to investigate the interactions between intestinal epithelial cells and the microbiota. The limitations and advantages of each model are summarised in Table 2. Some of these models have already demonstrated the ability to grow organoid-derived cells within the epithelial chamber, and others can accommodate them but with some optimisation required.

**Table 2.** Criteria to evaluate for selection of the appropriate organoid models to study the host-microbiota cross-talk in IBD

Model	Micro-injection	Apical-out organoids	Anaerobe Transwell	Anaerobic Gut-on-Chip	HuMiX
Epithelial layer: organoids?	Yes	Yes	Yes	Yes	No, but feasible
Differentiation	Crypt-villi	Crypt-villi	Monolayer	Crypt-villi	N/A
Mucus layer	Goblet cells [low expression]	Goblet cells [low expression]	Goblet cells [low expression]	Goblet cells [high expression]	Artificially added
Peristalsis	No	No	No	Yes	No
Type of inoculum [Single bacteria, complex community, metabolites]	Single, metabolites, complex [short term]	Single, metabolites	Single, metabolites, complex [short term]	Single, metabolite, complex	Single, metabolites, complex
Strict anaerobiosis feasible?	No	No	Yes	Yes	Yes
Direct contact between microbiota and epithelial cells?	Yes	Yes	Yes	Yes	No [separated by nonporous membrane]
Duration of the co-culture	<4 days	1 h	24 h	5 days	24 h
Possibility to add other cell types [eg, immune cells]	No	No	Short term in lower compartment	PBMCs, endothelial cells	CD4 + T cells
Outcome measures					
Integrity of the barrier	FITC-dextrans	FITC-dextrans	Transepithelial electrical resistance, FITC-dextrans	Cascade blue tracing	FITC-dextrans
Microbiota profiling [16S rRNA sequencing, proteomics..]	No	Yes	Yes	Yes	Yes
Sampling microbiota during co-culture	No	No	No	Yes	Yes
Organoid profiling [qPCR, RNA sequencing, western blot, microscopy, cytokine production]	Yes	Yes	Yes	Yes	Yes
Microscopy of the epithelium during co-culture	Yes	Yes	Yes	Yes	No
High-throughput?	Yes, provided specialised equipment	Yes	Yes	No	No
Availability and cost of the equipment used?	Low cost, wide availability	Low cost, wide availability	Low cost, medium availability	High cost, low availability	High cost, low availability

IBD, inflammatory bowel disease; FITC, fluorescein isothiocyanate; PBMC, peripheral blood mononucleat cells; NA,not available.

### 2.2. Microinjection of 3D organoids

3D organoids have closed structures, with the apical side of the epithelium, where most microbiota-epithelial interactions take place, projected inwards and therefore not very easily accessible. Different techniques can be employed to expose organoids to the microbiota, which include addition of metabolites, selected species of bacteria, or mixtures of complete patient-derived [filtered] faecal samples either by microinjection techniques or addition to the media.

Microinjection of microorganisms directly into the lumen of the differentiated 3D organoids has been employed in several laboratories, representing a useful technique to access the apical side of the epithelium.^68–70^ The lumen of organoids is characterised by hypoxic conditions.^42^ This facilitates the introduction of facultative or obligate anaerobic bacteria [Figure 1B], such as *Clostridium difficile,*^70^*Escherichia [E] coli,*^71^*E.coli* ECOR2,^72^ and faecal matter containing complex microbiota.^73^

The microinjection technique presents some limitations: a stable co-culture with obligate anaerobic bacteria cannot be sustained for a long period of time, due to the presence of small amounts of oxygen in the organoid lumen; it requires a very specialised set-up and training^73^; and leakage of injected bacteria towards the basolateral side can influence the readout. However, microinjection represents a very useful approach when studying facultative anaerobes and for high-throughput applications, especially when an automated set-up and appropriate training are available.^73^

### 2.3. 3D organoids with reversed polarity

Recently, the ability to grow reversed polarity [‘apical-out’] organoids has made the apical side of the epithelium easily accessible, making it possible to evaluate epithelial-microbe interactions by adding the microbes directly in the culturing medium,^74^ which is technically more convenient than microinjection [Figure 1B]. By understanding how matrix proteins control the epithelial [and thus organoid] polarity, researchers digested the matrix ‘bubble’, by using the chelator EDTA to disrupt divalent cation-dependent polymerisation of the ECM protein laminin. Upon continuation of the organoid culture in suspension using low-attachment plates, an inversion of the organoid polarity with the apical side on the ‘outside’ could be observed after only 3 days. These early ‘apical-out’ organoids continued to mature, including the different polarised differentiated cell types.^74^

This model has been used to study infection by invasive enteropathogens [*Salmonella enterica* serovar Typhimurium and *Listeria monocytogenes*], but could possibly be applied to study interactions with commensal microbes.^74^ The use of apical-out organoids represents an easily applicable option to investigate interactions with aerobic bacteria or bacterial metabolites, allowing multiple conditions to be easily tested in a high-throughput set-up. Yet, further validation is needed to assess the reliability of the readouts, as this technique does not guarantee a complete polarity reversion, making it difficult to distinguish between apical and basolateral interactions. Compared with basal-out organoids, the composition of apical-out organoids is skewed towards absorptive enterocytes. In the future, a thorough characterisation of how polarity reversion affects apical-out organoids phenotype, metabolism, and response to microbial challenge will be needed to understand critical similarities and differences between this model and self-organised and polarised organoids.^74^

### 2.4. Organoid-derived epithelial monolayers on Transwells

To make the apical side more accessible, another advance has been made by the linearisation of 3D organoids into 2D systems **[**Figure 1B**]**. Human small intestinal^75,76^ or colonic^77^ organoids created from patient biopsies can be cultured using the standard protocols,^52^ fragmented into small clumps of cells/single cells, and subsequently plated onto a extracellular matrix-coated dish-well or Transwell insert, to create an organoid-derived monolayer.^76,78,79^ Differentiation towards the different epithelial cell lineages occurs during the time period [± 7 days] of linearisation of the cells towards a strong, intact monolayer. Upon differentiation, the production of mucus by goblet cells is observed.^78^

In this model, the introduction of microorganisms is executed via addition to the culture media [Figure 2A].^78,80,81^ This model enables co-culture of organoids with aerobic bacteria and microbial-derived metabolites for several hours [<24 h for bacteria, <48 h for metabolites]^78,79,82,83^ in an easily applicable set-up, and enables comparison of several conditions in one experiment. However, co-culture with strictly anaerobic bacteria is not feasible in this particular model as the system is kept in aerobic conditions to guarantee the organoid survival.

### 2.5. The anaerobic Transwell model

Recently, various strategies have been developed to overcome this limitation by maintaining the apical chamber of a Transwell insert in an anaerobic environment, while keeping the basolateral chamber in aerobic conditions.^84–86^

In one model, the enteroid anaerobe co-culture [EACC] system, human jejunal organoid-derived monolayers grown on Transwell inserts were placed onto modified gaskets sealed in place using double-sided adhesive tape on a gas-permeable plate. When keeping the entire system in an anaerobic chamber, co-culture with the obligate anaerobic bacteria *Bacteroides thetaiotaomicron* and *Blautia sp.* could be sustained for at least 24 h.^84^ This cost-effective model has the advantages of enabling co-culture with anaerobic species for a limited time period [due to its static nature], and the possibility to employ in high-throughput experiments. However, it is not yet commercially available, and reproduction of the model could be time-consuming and would require optimisation.

In another model, the intestinal hemi-anaerobic co-culture system [iHACS], human colonic-derived epithelial monolayers are grown on Transwell inserts whose upper compartment is sealed off by a plug, enabling the co-culture with obligate anaerobe bacteria *Bifidobacterium adolescentis* and *Akkermansia muciniphila* for 24 h. Upon availability of the plug, the model can be implemented in every laboratory, although co-culture is also limited to 24 h.^85^

Finally, an alternative model has been developed where human colonic-derived epithelial monolayers are seeded within a micro-fabricated insert with tailored oxygen permeability properties, allowing the creation of an oxygen gradient between the luminal and basal compartments [0.8 ± 0.1% O_2_; 11.1 ± 0.5% O_2_]. Within this device, epithelial cells grown in the basolateral compartment polarise and remain viable during co-culture with facultative and obligate anaerobes *Lactobacillus rhamnosus*, *Bifidobacterium adolescentis*, and *Clostridium difficile* within the luminal compartment up to 24 h.^86^ The disadvantage of this model is that the culture area is relatively large [equivalent to a 12-well Transwell insert], thus requiring a substantial amount of starting material for the epithelial compartment.

## 3. Towards More Physiologically Relevant Organoid Models

Organoids are composed of epithelial cell types only.^87^ Depending on the proposed research question, this can be advantageous as it eliminates the confounding factor given by the presence of other cell types. This aspect is very important for IBD, in which defects in several epithelial cell type functions have been observed.^18^ However, for other questions, more ‘complex’ *in vitro* research models must include other cell types known to be essential in intestinal physiology and pathophysiology,^54,88^ including a functional immune system, enteric nerves, or nutrients providing the mesenchymal niche.^89^ Co-culture with immune cells [macrophages,^83^ neutrophils,^71^ T lymphocytes,^90^ and intraepithelial lymphocytes,^91^] as well as fibroblasts,^92^ adipocytes,^93^ and enteric nervous system cells,^94^ have been key to gain knowledge on the role of these cell types in host-microbial interactions in IBD.^95^

Maintaining the gut microbiota and host cells in a co-culture system is currently challenging. Host cells require an aerobic environment, whereas most members of the microbiota are facultative or obligate anaerobes, requiring an environment which is [virtually] oxygen-free. In addition, both host and bacterial cells require a specific medium supporting their growth and metabolism, resulting in a significant challenge to properly model microbiota host-interactions in the gastrointestinal tract.^53^

Furthermore, the static nature of culture conditions of organoids results in microbial overgrowth and potential damage to epithelial host cells due to nutrients and oxygen depletion, and accumulation of organic waste [eg, acetate or lactate]. So far, this issue has been tackled by keeping the microbial and epithelial culture compartments separate,^96^ or by using a short co-culture time [from half an hour to several hours], followed by bacterial elimination using bacteriostatic antibiotics.^97^ However, to properly mimic host-microbiome interactions *in vitro*, viable and functional intestinal tissue and microbial cells must be kept within the same confined space for a longer period of time.^98,99^ The introduction of fluid flows facilitating nutrient and oxygen uptake, and fluid shear stresses providing physiologically relevant mechanical signals to organoid cells, has represented a possible solution to tackle this problem.^100^

Organoids also lack peristalsis-like motion, which is important to stimulate *in vitro* intestinal differentiation, providing an improved *in vitro* model of the intestinal epithelium.^101,102^ Finally, organoid-derived monolayers lack the crypt-villus structure to properly mimic the host-microbial interactions in the gut; however, recent studies show that this organisation is an attainable goal.^103^ Another challenge is the replication of the mucus layer,^24^ including the small intestinal single layer and the colonic double layer composed of inner and outer mucus layers associated with different densities and distinct microbial communities.^104^

3D human intestinal organoids are able to recapitulate mucus production as observed *in vivo*. However, this is enclosed in the central lumen, making it difficult to investigate its role in host-microbiota interactions during IBD.^105^ Conversely, ileal and duodenal organoid-derived monolayers cultured on a Transwell only show the production of a thin mucus layer [<36 µm thick].^78,79^ Hence, these models are currently unable to reproduce the physiologically important bilayer structure seen in human colonic mucus.^105^

## 4. Microfluidic-based and Organoid-on-chip Models

The development of microfluidic devices incorporating organoid-derived cells has overcome some of these limitations, providing a more representative and physiologically relevant model to investigate host-microbiota interactions. Within these models, cells are cultured with organ-relevant spatiotemporal chemical gradients and dynamic mechanical cues, to reconstitute the structural tissue arrangements and functional complexity of the living organism *in vitro*.^106^ The use of these *in vitro* models to study host-microbe interactions has been previously reviewed.^107^

### 4.1. Gut-on-chip

Several ‘gut-on-chip’ devices have been developed.^99,108–110^ A gut-on-chip device consists of two channels simulating the gut lumen and a blood vessel, separated by an ECM-coated membrane and Caco-2 cells^99^ [Figure 1C]. In contrast to static cell monolayers or organoids, fluid flow and peristalsis-like deformations applied to the epithelial layer stimulate Caco-2 cells to differentiate into the four different types of intestinal epithelial cells, and to organise in villus-like structures.^111^ Endothelial cells and blood peripheral mononuclear cells can also be introduced in the lower channel, to obtain a more advanced system.^101^

However, gut-on-chip devices have some limitations, including the use of Caco-2 cells [as these cells are easier to introduce to the system and give rise to a confluent monolayer faster than organoids], and the ability to sustain the growth of multiple bacteria within the system only in a few cases^101^

### 4.2. Organoid-on-chip

Recently, 2D human-derived intestinal organoids have been introduced into gut-on-chip systems,^109^ combining the advantage of organoids [tissue differentiation] with those of gut-on-chip [controllable flow, mechanical cues, and tissue-tissue interaction]. In the microfluidic primary human intestine chip model [‘organoid-on-chip’], fragments from human duodenal organoids are plated onto an ECM-coated porous membrane, and primary human intestinal microvascular endothelial cells are seeded on the opposite side of the same membrane within a parallel channel. Similarly to the gut-on-chip model, fluid flow and peristalsis-like deformations promote epithelial multilineage differentiation and crypt-villi formation,^109^ with the final organoid monolayer mimicking epithelial functions [proliferation and response to infection] better than the original 3D organoid.

One drawback of gut-on-chip and organoid-on-chip devices is the presence of an aerobic environment within the epithelial chamber, which prevents the introduction of strictly anaerobic bacteria,^99,101^ thus failing to represent bacterial species that play a major role in the gut.^107,112^

This limitation has been addressed thanks to the development of a novel microfluidic device with a transluminal hypoxia gradient, which allows study of the effect of a complex living human gut microbiome [including obligate anaerobes] on the epithelium.^113^ This anaerobic human gut-chip was used to successfully co-culture a fresh gut microbiome isolated from human infant stool samples with a human ileal organoid monolayer. The presence of a hypoxia gradient within this system helped to sustain a physiologically relevant level of microbial diversity, with ratios of Firmicutes and Bacteroidetes similar to those observed in human faeces, and an extended co-culture time [up to 5 days]. Collectively, this allows better modelling of the physiological interactions between the intestinal epithelium and the anaerobic gut microbiota.^113^

Another adaptation of the human gut-on-a-chip model is represented by the anoxic-oxic interface-on-a-chip [AOI Chip]. This device contains a controlled gradient of oxygen thanks to the flow of both oxic and anoxic media in the chip, and allows the co-culture of Caco-2 epithelial cells and the anaerobic *Bifidobacterium adolescentis* and *Eubacterium hallii,* for up to 1 week.^114^

Overall, organoid-on-chip systems have the advantage of enabling long co-cultures of a complex microbiota culture in direct contact with organoid-derived cells. In addition, these models support spontaneous goblet cell differentiation and accumulation of a mucus bilayer structure with impenetrable and penetrable layers, and a thickness similar to that observed in the human colon, while maintaining a subpopulation of proliferative epithelial cells.^115^ However, these models are expensive, require specific training to use, and do not allow for high-throughput experiments. In addition, the anaerobic human gut-on-chips are not commercially available yet in the set-up used.

### 4.3. The human-microbial cross-talk module

Within the human-microbial cross-talk module [HuMiX], anaerobic bacteria can be maintained in an [almost] anoxic compartment^116^**[**Figure 1C**].** This device is composed of three parallel microfluidic chambers [microbial, epithelial, and perfusion chamber] separated by semipermeable membranes, including specific controllable inlets for each chamber.^107,116,117^ Currently, experiments have been performed with Caco-2 cells, but the implementation of organoids in the model is feasible without any further adaptations. Caco-2 cells are cultured for 7 days under continuous basal perfusion, followed by bacterial inoculation into the microbial chamber for 24 h co-culture in anaerobic sheer-free conditions. Following co-culture, the modular design of HuMiX allows easy disassembly and cell collection for detailed downstream analyses.^116^

Using this system, Caco-2 cells have been successfully co-cultured with the anaerobic bacteria *B.caccae* and *L.rhamnosus*,^116^ as well as immune cells [CD4 + T cells].^116^ In the future, this system will be further developed to become the so-called ‘immuno-HuMiX’, which will allow the coexistence of patient-derived microbiota, epithelial cells, and immune cells (eg, peripheral blood mononuclear cells [PBMCs]).

In HuMiX, the mucus-coated membrane separating the microbial and epithelial compartments enables the study of host-metabolite interactions but prevents the direct host-microbe contact. In addition, gut peristalsis and differentiation of the epithelium into various cell types is not present.^109,118^ As mentioned above, the introduction of organoids is feasible but would require a significant amount of starting material, given the large seeding surface [8 cm^2^], which would limit high-throughput experiments. As an advantage, multiple readouts [RNA sequencing/qPCR, immunostaining, western blot] are feasible within one device. Finally, the current ‘microbial microchamber’ is not completely devoid of oxygen, making this model less physiologically relevant and hindering the co-culture of strict anaerobes.^116^

## 5. Limitations of Current Organoid Models for Host-microbiota Studies and Future Challenges

Organoid technology is constantly evolving, and many challenges associated with these models need to be addressed in the near future. The economic burden of high-throughput experiments and required [commercially available] equipment for the different devices are still limiting factors.

The polymeric material [PDMS] used in gut-on-chips can often limit the applicability to drug studies by absorbing small molecules, due to its hydrophobic nature.^119,120^ Alternative materials or surface-coating techniques are being investigated to tackle these issues in the future.^121–124^ The 3D nature and structural complexity of organoids and organoids-on-chip also present clear challenges to microscopy imaging and image analysis. Methods to enable precise positioning and confinement of organoids,^125^ tissue-clearing methods during staining protocols,^126^ or the use of advanced 3D imaging techniques,^127^ are being developed to tackle these issues.

To reduce the overall variability of organoid experiments and related read-outs, standardisation of organoid culturing methods, including the used expansion and differentiation media, is paramount. Intestinal organoid culture medium is commercially available [IntestiCult™ Organoid Growth Medium, Stemcell Technologies], but due to its relatively high cost, the majority of the research laboratories are producing growth factors themselves [Wnt3A, Noggin, R-Spondin], which may cause batch-to-batch variation and differences in the cultivation and differentiation state of organoids. In addition, enrichment for a particular or rare cell type should be attainable in a standardised way.^128–130^ The Transwell model enables differentiation into the different lineages but maintains a linearised structure of the epithelium while microfluidic flow drives the cells into more physiological crypt-villi structures.^78,109^

Recreating the optimal co-culture conditions to keep the bacterial community representative of the starting composition and stable over time, while maintaining epithelial or immune cells viability, still remains a challenge. Even without co-culture with epithelial cells, the long-term culture of microbiota supporting both the metabolism and the composition of this complex community is currently challenging. The targeted culturing of selected species is feasible but, when culturing complete microbiota, the selection of a specific medium and culture conditions will inevitably favour the growth of certain phyla.^131^ In addition, bacterial media often contain components that damage mammalian cells,^132^ leading to necessary compromises in terms of the best medium to be used during the co-culture.

Furthermore, although IBD organoids derived from inflamed regions maintain disease- and patient-specific characteristics, they lose their acute inflammatory phenotype during organoid culture.^133,134^ Previous studies have shown a differential response of epithelial cells to butyrate in the presence/absence of inflammation,^82^ showing the need to unravel host-microbiota interactions with and without an acute inflammatory state, reflecting active and quiescent disease, respectively. Interestingly, a recent study has shown that exposure of organoids to a defined inflammatory mix is able to re-induce the inflammatory phenotype while maintaining patient specificity.^134^

## 6. Future D irections

### 6.1. The next generation co-culture model

Currently available models to study host-microbiota interactions in IBD are still not optimal and require compromising between models supporting high-throughput experiments, or those better representing the *in vivo* situation but limited in throughput and convenient handlings [Table 1]. To be able to study interactions with the microbiota, there is great need to develop a model that enables an aerobe-anaerobe interface in a commercially available setting, and can support high-throughput applications at the same time.

This would first of all require the presence of aerobe and anaerobe compartments, in which the direct interactions between a complex microbiota community and epithelial cells can be evaluated during long-term co-culture experiments. This can either be done by creating two separate compartments and performing co-cultures in an anaerobe environment, or by providing each compartment with the desired aerobic/anaerobic source.

Simultaneously, the presence of a representative mucus layer, with small intestinal organoids composed of single layer and colonic organoids composed of inner and outer mucus layers with different densities, would be essential to correctly mimic the *in vivo* situation.^24,104^ In addition to mucin-like formulations, hydrogel-based materials^135^ with enhanced functionalities [ie, spatial-temporal control of characteristics] will be more frequently used in future versions of organoid-on-chip systems, as they can be tailored to match some of the relevant properties and functions of intestinal mucin for specific applications.^136^

Furthermore, the presence of an oxygen gradient will be important to allow the establishment of separate microbial communities of mucosal and luminal microbiota along the direction of the oxygen gradient, which would reflect conditions closer to the *in vivo* situation.

Beyond the correct oxygen gradient and mucus layer, studies have highlighted the crucial importance of the *in vivo*-like intestinal tissue microenvironment in shaping the composition of the microbial community, as demonstrated by the mucus degrading-genus *Akkermansia,* which is found in great abundance within anaerobic gut-on-chips, but not in liquid cultures under the same oxygen and mucus conditions.^113^ Growing organoids on structured surfaces such as polymeric microscaffolds will be key to better mimicking the native *in vivo*-like microenvironment of the gastrointestinal tract, including the crypt-villi architecture.^137^ In this regard, the development of finly tuneable biomaterials, such as [functionalised] hydrogels, will be paramount in providing a better simulation of the extracellular matrix and the mechanics of soft tissues while supporting cell adhesion and protein sequestration,^138,139^ also addressing the issues related to poorly defined compositions and batch-to-batch variability of organoid matrices used so far.^140–142^

Real-time evaluation of cell organisation and structure to monitor monolayer integrity and cell viability during organoid differentiation and exposure to the microbiota, in addition to *ad-hoc* fine tuning of flow speed and oxygen concentrations, will also be key. To reduce the costs of the model [culturing of organoid cells, required media, and other components] the seeding surface should be limited, but enough to enable continuous sampling of the medium and allow several read-outs [RNA sequencing, proteomics, metabolomics, immunohistochemistry,..] of both microbial and host cells in each device.

The inclusion of several microsensors that can precisely monitor alterations over the complete time course of the experiment [oxygen concentrations, barrier integrity measurements, temperature, oxygen, pH, integrity, flow] are of major interest, together with regular, easily accessible sampling of the different compartments [epithelial cells, microbiota].^143^

To complement the model, co-culture with other cell types including separate medium compartments should be optimised. In combination with patient-specific epithelial cells and microbiota, the addition of immune cells brings these co-culture models to the next level for evaluation of patient-specific responses or preclinical testing of new compounds.

The implementation of advanced [microfluidics] organoid-based models not only requires specialised knowledge and training, but also involves substantial implementation costs. To this end, the development of automated systems capable of performing several standardised read-outs will be key to achieving the successful introduction of these systems in daily laboratory practice. In addition, this will also help reduce the experimental variability and allow comparison of experimental results among different groups, especially when it comes to preclinical testing of compounds or microbiota mixtures.

### 6.2. Applications

Organoids and organoid-derived models are amenable to various applications **[**Figure 2B**]**. These can be combined to understand the role of host-microbiota interactions in IBD pathogenesis, and aid in finding microbial interventions tailored to the single individual, resulting in better prevention strategies and treatment success.

#### 6.2.1. ’Omics and single-cell approaches

The different models reviewed are amenable to ’omics data generation, which can be helpful to interrogate the role of the epithelial genetic [patient-derived or engineered] or epigenetic background [acquired from the persistent exposure to pro-inflammatory signals].^58,144^ 3D organoid models and organoid monolayers on Transwells are more suited for generating a single type of ’omics readout in many different samples, whereas microfluidics systems such as HuMIX allow multiple ’omics readouts to be produced from the same sample in a selected number of conditions.

Gut microbes and their metabolites affect each intestinal epithelial subtype differently. To evaluate cell-specific responses, organoid differentiation can be skewed towards a specific lineage [eg, Paneth cells or goblet cells] before exposure to the bacterial mixture.^128^ Alternatively, techniques such as fluorescense activated cell sorting [FACS]^145^ or mass cytometry^146^ may allow the isolation of cells from organoids after exposure to microbiota, similarly to what has been achieved for mouse intestinal tissue.^147^

Recently, single-cell ’omics approaches have been applied to characterise individual cells in mouse organoids,^148^ or organoids derived from active CD lesions,^149^ and to investigate the differential response of intestinal cell types to bacterial infection.^150^ In the future, a similar approach could be applied to provide new insights into the mechanisms by which the microbiota or specific probiotics may influence the function of specific subpopulations of cells in the gut in relation to IBD.

#### 6.2.2. Personalised disease models

CRISPR/Cas9 genome editing technology has been employed to develop genetically defined cultures from organoids.^151–154^ The manipulation of specific genes linked to genetic susceptibility could help gain a better understanding of disease mechanisms, also in relation to microbial exposure [eg, *GATM*, *NOD2*, *HNF4A*, *ATG16L1*,… ]].^155^ Such models could be employed to dissect the role of epithelium in early-onset IBD, where a specific genetic mutation of the intestinal epithelium causes a defect in barrier function^156^ and no inflammation has developed yet. Organoids enriched for a specific cell type^157^ can further help elucidate the role of specific epithelial subpopulations in modulating the adhesion of IBD-associated bacterial strains, such as the recently discovered sentinel goblet cells.^158^

Co-culturing IBD-derived and matched healthy organoids in parallel, followed by addition of patient-derived microbiota and immune cells, could also throw some light onto whether the loss of tolerance towards the microbiota is a cause or consequence of the disease, a fundamental question in IBD research.

#### 6.2.3. Drug/microbial screening

Organoid biobanks can be established from patient-derived organoids. One example is the recently established biobank containing IBD-derived organoids from various parts of the gastrointestinal tract.^159^ By employing high-throughput approaches,^160,161^ these can be used to screen microbial species or their metabolites.^78,162–164^ Because the screening is performed in subpopulations of organoids, such approaches are key when studying heterogeneous diseases such as IBD,^165–167^ as they reduce the variability and allow precision medicine approaches. By doing so, patient treatments could be optimised before application, reducing treatment failure. The introduction of patient-derived or engineered organoids within microfluidics devices also helps re-create a better *in vivo*–like disease phenotype,^168^ thereby facilitating the development of robust disease models for improved personalised drug screening and matching.^169,170^ Overall, organoids are useful for target identification and validation in the early stages of drug discovery, thanks to their similarity with actual organs. Instead, organs-on-a-chip are more suited for subsequent efficacy and safety screening, as they provide a more reproducible and controllable environment.^171^

#### 6.2.4. Personalised medicine and microbial therapies

Microfluidics devices can be employed to couple patient-derived intestinal organoids with host-specific isolated immune cells, PBMCs, or microbiota [faecal inocula or individual-specific isolates], allowing the establishment of models amenable to personalised medicine approaches.^172^ For instance, these models could be exploited to define how different commensal microorganisms within a patient-specific microbiota community contribute to IBD pathophysiology.^173^ On the one hand, these systems could help gain a better understanding of the causal relationship between the abundance of each individual genus of bacteria and the distinct functions of the co-cultured human intestinal epithelium. On the other hand, these systems could be used to unravel how a dysfunctional IBD-derived epithelial layer could impact on the diversity and composition of the microbiota, favouring the growth of specific pathogenic bacteria [eg, pathogenic *E. coli*], while reducing beneficial ones [eg, *Faecalibacterium prausnitzii*]. Overall, the integration of these approaches could result in the development of personalised microbial therapies, including microbiome-based therapeutics such as genetically engineered commensal bacteria,^174^ or to assess the efficacy of FMT in a patient-specific manner.^113^

## 7. Concluding remarks

In recent years, the fundamental role played by host-microbiota interactions in IBD has begun to be unravelled. However, further research needs to be carried out to understand whether it will be possible to resolve dysbiosis, and which elements of the microbiota and species are essential to do this. Moreover, it is likely that each microbial therapy will need to be tailored for each individual.

Intestinal organoids, alone or within a microfluidics system, have shown the potential to become the gold standard in the study of host-microbiota interactions in IBD in a patient-specific manner. However, so far studies that analytically compare organoid-based systems and benchmark them for their suitability to look at host-microbiota interactions in IBD have been lacking. In this review, we have presented a variety of models, from 3D organoids and 2D organoid-derived monolayers to organoid-on-chip systems, and discussed their advantages, limitations, and suitability for different research questions and applications.

In the future, the use of organoid models, in combination with the patient’s specific microbiota composition and immune cells, will help to provide a better understanding of disease onset mechanisms and to identify the key bacterial species required for resolving dysbiosis. This understanding could lead to more targeted microbial therapies, which either alone or in combination with traditional approaches have the potential to revolutionise IBD management and ultimately improve patient outcomes.
